# Estrogen inhibits starvation‐induced apoptosis in osteocytes by a redox‐independent process involving association of JNK and glutathione S‐transferase P1‐1

**DOI:** 10.1002/2211-5463.12216

**Published:** 2017-04-05

**Authors:** Vladana Domazetovic, Filippo Fontani, Gemma Marcucci, Teresa Iantomasi, Maria Luisa Brandi, Maria Teresa Vincenzini

**Affiliations:** ^1^ Department of Biomedical, Experimental and Clinical Sciences “Mario Serio”(Biochemistry section) University of FlorenceItaly; ^2^ Department of Surgery and Translational Medicine (Endocrinology Section)University of FlorenceItaly

**Keywords:** estrogen, GSTP1‐1 expression, JNK activity, osteocyte apoptosis, RANKL/OPG ratio

## Abstract

Estrogen deficiency causes bone loss as a result of microdamage, oxidative stress, and osteocyte apoptosis. A relationship between oxidative stress‐induced apoptosis, c‐Jun N‐terminal kinase (JNK) activation, and expression of factors involved in bone remodeling has been demonstrated in osteocytes. However, the molecular regulation of these events in osteocytes treated with 17β‐estradiol (17β‐E2) remains unexplored. The MLO‐Y4 murine osteocyte‐like cell line was used as a model to study starvation‐induced apoptosis and ROS production during 17β‐E2 treatment. Expression of glutathione S‐transferase P1‐1 (GSTP1‐1), receptor activator kB ligand (RANKL), osteoprotegerin (OPG), sclerostin, and kinases activation were measured by western blot. In addition, the GSTP1‐1/JNK association was assessed by immunoprecipitation, and GSTP1‐1 involvement in the osteocyte response to 17β‐E2 was detected by specific siRNA transfection. 17β‐E2 prevents starvation‐induced apoptosis (DNA fragmentation and caspase activation), the increase in sclerostin expression and the RANKL/OPG ratio, which are all related to JNK activation due to oxidative stress in osteocytes. This occurs through GSTP1‐1 overexpression, which can inhibit JNK activation by formation of a GSTP1‐1/JNK complex. No early antioxidant action of 17β‐E2 has been found but the estrogen effect is similar to N‐acetylcysteine which, by increasing the intracellular redox state, maintains JNK bound to GSTP1‐1. Thus, the antiapoptotic and osteogenic effect of 17β‐E2 in MLO‐Y4 occurs by a redox‐independent process involving GSTP1‐1/JNK association. This study clarifies at molecular level the effect of 17β‐E2 on osteocyte activity and identifies a possible role of GSTP1‐1 and JNK activity in bone remodeling and repair mechanisms.

**Abbreviations**
17β‐E2: 17β‐Estradiol
AEBSF: 4‐(2‐aminoethyl)‐benzenesulfonylfluoride
DPI: diphenyleneiodonium
ERK1/2: extracellular signal‐regulated kinase
GSTP1‐1: glutathione S‐transferase P1‐1
JNK: c‐Jun N‐terminal kinase
Mito TEMPO: 2‐(2,2,6,6‐Tetramethylpiperidin‐1‐oxyl‐4‐ylamino)‐2‐oxoethyl‐triphenylphosphonium chloride monohydrate
MKP‐1: protein kinase phosphatase‐1
NAC: N‐acetylcysteine
OPG: osteoprotegerin
RANKL: receptor activator kB ligand
ROS: reactive oxygen species
scr siRNA: scrambled siRNA

Estrogens influence the size and shape of the skeleton during growth and contribute to bone homeostasis during adulthood. The decline in estrogen levels, associated with menopause, causes bone loss in women related to a high rate of bone remodeling 1, 2 which results from the close balance between osteoblast and osteoclast activity, leading to either bone formation or bone resorption, respectively. Recently, new data support the central regulatory role of osteocytes in the maintenance of this balance and thus in viability and functionality of bone 3, 4. Estrogen deficiency alters osteoblast mineralization and osteoclast resorption activity, leading to increased bone resorption and osteoporotic cascade 1, 2, 5. In fact, estrogens, through their receptors, increase bone mineralization 1, 2, 6, 7, whereas estrogen deficiency causes an increase in osteoblast and osteocyte apoptosis in both the trabecular and cortical compartment 1. This causes a deteriorated microarchitecture and reduced mechanical strength of bone as well as increased fracture risk 1, 8. The marked reduction in estrogen induces oxidative stress related to menopause and bone loss 9, 10, 11, 12. In fact, it has been demonstrated that, in addition to aging, ovariectomy induces oxidative stress in rat femurs along with decreased activity of antioxidant systems 12. Moreover, the protective effects of estrogens on bone result from their ability to attenuate oxidative stress 13 by activating antioxidant gene expression 9, 10, 11, 13 or by a direct free radical scavenging activity 14. However, other data indicate that estrogen therapy has no significant effect on oxidative stress levels 11, 15.

Many studies, performed on osteoblasts and osteoclasts, have linked reactive oxygen species (ROS) and antioxidants to bone metabolism and bone remodeling 13, 16, 17, 18. In osteocytes, high levels of ROS induce increased apoptosis 14, 19, 20, 21, and this is also related to loss of estrogens and a decrease in bone mineral density 12, 22, 23, as occurs in aging and chronic glucocorticoid treatment 12, 19, 22, 24, 25, 26, 27. Since osteocytes are mechanosensitive cells 3, 4, increased apoptosis may impair the ability of bone to adaptively respond to mechanical loading and to repair microdamage due to physiological or pathological events such as aging, osteoporosis, or osteoarthritis, also related to oxidative stress 3, 19, 22, 23, 24, 25, 26. It has been demonstrated that the modulation of mechanical loading and ovariectomy induce an increase in osteocyte apoptosis and lead to microdamage 19, 22, 23 with subsequent osteoclastic invasion to the damaged site and activation of the remodeling process 28, 29, 30, 31. Estrogen treatment prevents osteoblast and osteocyte apoptosis 1, 2, 5, 13, 14, 23, although molecular mechanisms and biochemical signals, by which estrogens protect osteocytes from the apoptosis due to microdamage and/or oxidative stress, have not been fully elucidated. Similarly, to our knowledge in osteocytes, no data are reported on the molecular processes by which estrogens modulate the expression of cytokines involved in bone remodeling in the presence of oxidative stress‐induced apoptosis.

The aim of this study was to investigate the molecular processes by which 17β‐Estradiol (17β‐E2) prevents osteocyte apoptosis and the abnormal expression and release of factors related to increased oxidative state and bone remodeling. The effect of 17β‐E2 on ROS production and the activation of redox‐regulated kinases, such as extracellular signal‐regulated kinase (ERK1/2) and c‐Jun N‐terminal kinase (JNK), were also studied. Indeed, in osteocytes, these kinases are involved in oxidative stress‐induced apoptosis and expression of receptor activator kB ligand (RANKL), osteoprotegerin (OPG), and sclerostin 21. To clarify the molecular action of estrogen on these events, the role of mitogen‐activated protein kinase phosphatase‐1 (MKP‐1) and glutathione S‐transferase P1‐1 (GSTP1‐1), both involved in the regulation of JNK activity, were investigated 32, 33, 34, 35. This study was performed in MLO‐Y4, a murine osteocyte‐like cell line, that constitutes an *in vitro* model for studying osteocyte viability and apoptosis in response to microdamage and bone diseases 31, 36, 37, 38. Apoptosis due to oxidative stress was induced in MLO‐Y4 by serum starvation 21. This method causes apoptosis not due to proinflammatory and proapoptotic factors and mimics *in vitro* a metabolic condition of oxidative stress that may be similar to what occurs *in vivo* in the bone environment after microdamage 19, 20, 31, 37, 38, 39. In fact, apoptosis due to microdamage may be related to a disruption of blood and fluid flow with consequent lack of various endocrine factors, including estrogens 31, 40, 41. Thus, it is possible to investigate “*in vitro*” the regulatory role of estrogens in osteocyte apoptosis in bone remodeling.

## Materials and methods

### MLO‐4Y culture and treatment

MLO‐4Y (a gift from L. Bonewald, University of Missouri‐Kansas City) were cultured at 37 °C in a 5% CO_2_ humidified atmosphere in alpha‐MEM medium supplemented with 5% calf serum (GE Healthcare HyClone, Little Chalfont, UK), 5% FBS (HyClone), 2 mm L‐glutamine, 72 mg·L^−1^ (L)‐penicillin, and 100 mg·mL^−1^ (L)‐streptomycin (complete medium). Cells at 70–80% confluence were treated for 30 min in a medium, in which FBS was substituted with charcoal stripped FBS (Sigma‐Aldrich, Saint Louis, MO, USA), with 17‐β‐E2 (Sigma‐Aldrich) at various concentrations (1, 10 or 100 nm), or in complete medium in the absence (untreated cells) or in the presence of 5 mm *N*‐acetylcysteine (NAC, Sigma‐Aldrich) for 16 h. Subsequently, after removal of the media, treated cells were cultured for another 4 or 24 h in serum free medium in the presence of 17‐β‐E2 or NAC. Untreated cells were cultured for 4 or 24 h in serum free medium (S, starved cells) or in fresh complete medium (C, control). 0.01% ethanol (vehicle for estrogen) was added to all untreated 17‐β‐E2 cells. For experiments with inhibitors, cells were pretreated for 30 min in complete medium with 1 μm diphenyleneiodonium (DPI) or 100 μm 4‐(2‐aminoethyl)‐benzenesulfonylfluoride (AEBSF) or 5 nm 2‐(2,2,6,6‐Tetramethylpiperidin‐1‐oxyl‐4‐ylamino)‐2‐oxoethyl‐triphenylphosphonium chloride monohydrate (Mito TEMPO) (Sigma‐Aldrich), in the presence or in the absence of estrogen. Subsequently, the complete medium was removed and for another 4 or 24 h, cells were cultured with inhibitors with or without estrogen in serum‐free medium. In experiments with DPI, 0.003% DMSO was added to DPI untreated cells. Some treatments were performed in cells transiently transfected with 60 nm mouse GSTP1‐1 siRNA corresponding to two DNA target sequences of mouse GSTP1‐1 (5′‐CCCUCAUCUACACCAACUA[dT][dT]‐3′); 5′‐UAGUUGGUGUAGAUGAGGG[dT][dT]‐3′) (Sigma) or scrambled siRNA (scr siRNA) (Universal Negative Control #1, Sigma), using lipofectamine RNAiMAX™ (Invitrogen Carlsbad, CA, USA) according to the manufacturer's protocol. The ability of siRNA to silence the expression level of GSTP1‐1 mRNA was checked 24 h after transfection.

### Measurement of MLO‐4Y apoptosis

MLO‐4Y seeded in six‐well plates and treated with 17‐β‐E2 as above reported were used to assess apoptosis using the Cell Death Detection ELISA plus Kit (Roche Laboratories, Nutley, NJ, USA), according to the manufacturer's instructions. The apoptosis assay was performed measuring the specific increase in mono‐ and oligonucleosomes released into the cytoplasmic fractions obtained from 10^4^ cells using the following formula: fold‐increases = absorbance of the samples/absorbance of the corresponding controls (C).

### Measurement of intracellular ROS

The intracellular ROS production was measured in MLO‐4Y seeded in six‐well plates and treated as above reported. One hour before the end of treatments, 2′,7′‐dichlorodihydrofluorescein diacetate (H2DCFDA, Invitrogen) was added to culture medium. After PBS washing, cells were lysed in RIPA buffer (50 mm Tris/HCL pH 7.5, 1% Triton X‐100, 150 mm NaCl, 100 mm NaF, 2 mm EGTA; Sigma‐Aldrich), centrifuged at 11 600 ***g*** for 10 min, and analyzed immediately by fluorescence spectrophotometric analysis at 510 nm. Data, normalized on total protein content, were expressed as fold‐increase over the control values.

### Western blot analysis

The phosphorylation of ERK1/2 and JNK, the activation of caspase‐3 and the expression of RANKL, OPG, sclerostin, GSTP1‐1, and MKP‐1 were performed by western blot in MLO‐4Y treated as above reported. Cells were lysed in ice cold RIPA buffer containing phosphatase and protease inhibitor cocktails (Sigma) and centrifuged at 11 600 ***g*** for 10 min. Equal amounts of total proteins (40–60 μg) from whole‐cell extract were subjected under reducing conditions to SDS/PAGE on 10% gel and electrotransferred to PVDF membrane (GE Healthcare). Proteins were visualized by incubating the membranes with specific primary antibodies: anti‐caspase 3 or anti‐phospho‐ERK 1/2 or anti‐phospho‐JNK (Cell Signalling Technology, Beverly, MA, USA), or anti‐RANKL or anti‐OPG or anti‐sclerostin or anti‐GSTP1‐1 or anti‐MKP‐1 (Santa Cruz Biotechnology, Inc., Dallas, TX, USA). Subsequently, membranes were stripped and reprobed with anti‐ERK1/2 or anti‐JNK or anti‐β‐actin for normalization and densitometric analysis. Secondary antibodies conjugated to horseradish peroxidase were used to detect antigen–antibody complexes with a chemiluminescence reagent kit (Bio‐Rad, Hercules, CA, USA). image j software (National Institutes of Health, Bethesda, MD, USA) was used to perform quantitative analyses, and band values were expressed as percentage relative to values of control.

### Immunoprecipitation and immunoblot

Immunoprecipitation was carried out using 2 μg of anti‐GSTP1‐1 or anti‐JNK antibody or negative control mouse IgG (BIOCARE Medical, Pacheco, CA, USA) and 400 μg of total protein lysates, for 16 h at 4 °C. Subsequently, protein A/G PLUS‐Agarose Immunoprecipitation Reagent (Santa Cruz Biotechnology, Inc.) was added (20 μL) for 1 h at 4–5 °C, after which washes were carried out in PBS supplemented with Tween‐100 (0.5%). Precipitate was suspended in sample buffer without β‐mercaptoethanol (nonreducing conditions) and was subjected to SDS/PAGE (10%) followed by electrotransfer to PVDF membrane. Reaction with the anti‐JNK or anti‐GSTP1‐1 overnight at 4 °C was carried out. Subsequently to stripping, membranes were reprobed with anti‐GSTP1‐1 or anti‐JNK or anti β–Actin.

### Measurement of RANKL and OPG release

Receptor activator kB ligand and OPG release was measured in the culture medium of MLO‐Y4 treated as above reported, using quantitative sandwich enzyme immunoassay kits (R&D System, Minneapolis, MN, USA) according to the manufacturer's instructions. Data, normalized on total protein content, were expressed as fold‐increase over the control values.

### Protein assay

Protein concentrations were determined by the bicinchoninic acid solution protein reagent assay (Pierce, Rockford, IL, USA) using bovine serum albumin as the standard (Sigma‐Aldrich).

### Statistical analysis

All experiments were performed four times in triplicate. Data were expressed as mean ± SEM and statistical significance of the differences was determined by Student's *t*‐test. *P* ≤ 0.05 was considered statistically significant.

## Results

### Effect of 17 β‐E2 on apoptosis and ROS production in starved MLO‐Y4 cells

**Figure 1:**
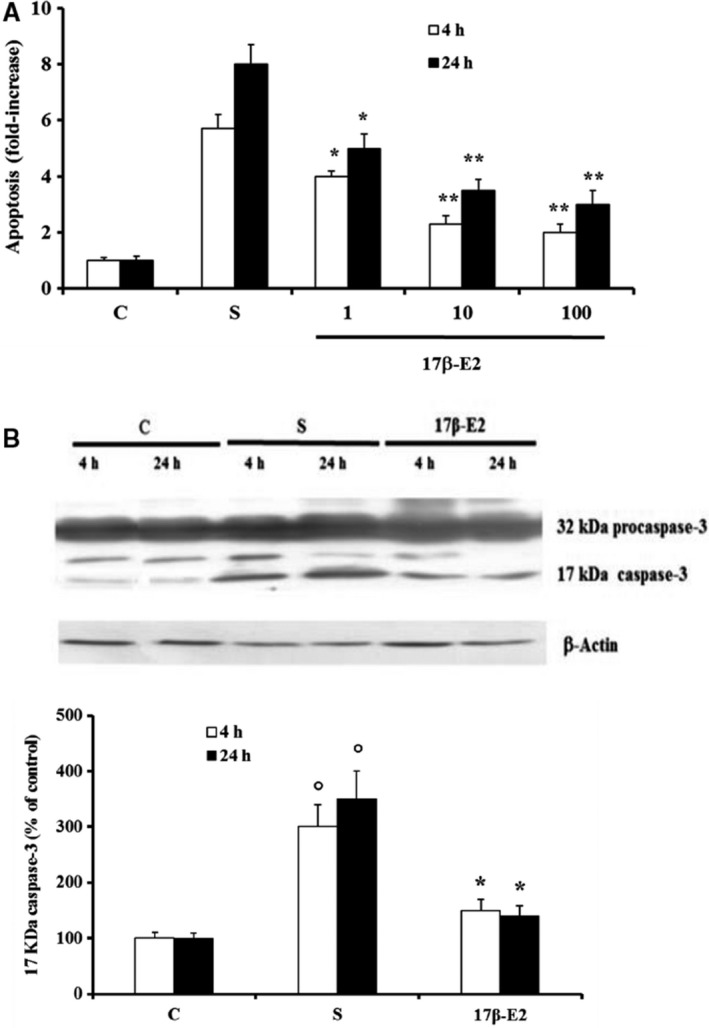
Effect of various concentrations of 17β‐E2 on apoptosis and active 17 KDa caspase‐3 in MLO‐Y4 cells. Apoptosis (A) and active 17 KDa caspase‐3 (B) were measured in MLO‐Y4 cells cultured for 4 h and 24 h in complete medium (C, control) or in serum‐free medium (S, starved cells). S were treated or not with 17β‐E2 as reported in Materials and methods. Apoptosis data, relative to mono‐ and oligonucleosomes released into the cytoplasmic fraction from 10^4^ cells treated with various nm concentrations of 17β‐E2, are expressed as fold‐increase over the respective C values and are the mean ± SEM of four experiments. Active 17 KDa caspase‐3 was measured in cells treated with 10 nm 17β‐E2 by Western blot analysis. Blots are representative of four experiments and the active 17 kDa caspase‐3 values are normalized with β‐Actin bands obtained by densitometric analysis and reported as the mean percentage ± SEM relative to the respective C values in the bottom. *P ≤ 0.05 and **P ≤ 0.005 compared to the respective S values; °*P* ≤ 0.005 compared to the respective C values.

**Figure 2:**
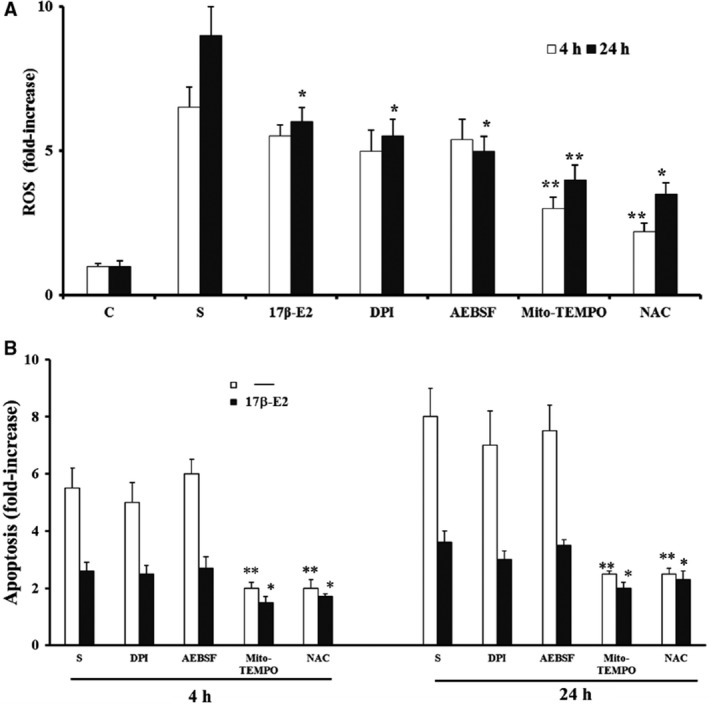
Effect of 17β‐E2, DPI, AEBSF, Mito Tempo and NAC on intracellular ROS and apoptosis in MLO‐Y4 cells. Intracellular ROS (A) and apoptosis (B) were measured in MLO‐Y4 cells cultured for 4 and 24 h in complete medium (C, control) or in serum‐free medium (S, starved cells). S were treated or not with 10 nm 17β‐E2, 1 μm DPI, 100 μm AEBSF, 5 nm Mito‐TEMPO, or 5 mm NAC as reported in Materials and methods. ROS data, normalized on total protein content, and apoptosis data, relative to mono‐ and oligonucleosomes released into the cytoplasmic fraction from 10^4^ cells, are expressed as fold‐increase over the respective C values. The data are the mean ± SEM of four independent experiments. *P ≤ 0.05 and **P ≤ 0.005 compared to the respective S values.

Figure 1A shows that apoptosis increased remarkably after 4 and 24 h from the starvation, and the pretreatment of MLO‐4Y for 30 min with various concentrations of 17 β‐E2 reduced starvation‐induced apoptosis at both times, as compared to the respective untreated starved cells**.** Under the experimental conditions in this study, the maximum effect of apoptosis inhibition (about 60%) was measured at 10 nm 17 β‐E2, a concentration near physiological value and used in the subsequent treatments. No statistically different change between 10 nm and 100 nm 17 β‐E2 was measured. The antiapoptotic effect of 10 nm 17 β‐E2 was confirmed by the decrease of caspase‐3 active form involved in the apoptosis of osteocytes. In fact, estrogen significantly decreased—by about 50–60%—active 17 kDa caspase‐3, that derives from proteolytic cleavage of inactive 32‐kDa procaspase‐3 14, 21, 42 (Fig. 1B). Figure 2 shows that the levels of ROS increased after 4 and 24 h from the starvation, and 17 β‐E2 lowered ROS content partially (about 30%) only after 24 h, as compared to untreated starved cells. Subsequently, 17 β‐E2 effect on ROS and apoptosis was compared with that obtained in cells treated with: DPI or AEBSF, inhibitors of NADPH oxidase activity; Mito TEMPO, a specific scavenger of mitochondrial ROS; or NAC, a direct antioxidant 14, 21, 43, 44, 45 (Fig. 2A,B). The inhibitors were used at concentrations able to inhibit ROS production. Under our experimental conditions, AEBSF and DPI, similarly to 17 β‐E2, significantly reduced ROS levels by about 40% only after 24 h; conversely, Mito TEMPO significantly inhibited ROS levels by about 50–60% at both times, similarly to NAC (Fig. 2A). Figure 2B reports the apoptosis levels measured in cells treated with inhibitors and stimulated or not by 17 β‐E2; no change in apoptosis was observed in DPI‐ and AEBSF‐treated cells at either studied time and no synergic effect was detected in cells treated with these inhibitors plus estrogen. Mito TEMPO and NAC decreased apoptosis levels by about 60% as compared with starved cells stimulated or not with estrogen (Fig. 2B).

### Effect of 17 β‐E2 on ERK1/2 and JNK activation and MKP‐1 expression in starved MLO‐Y4 cells

**Figure 3:**
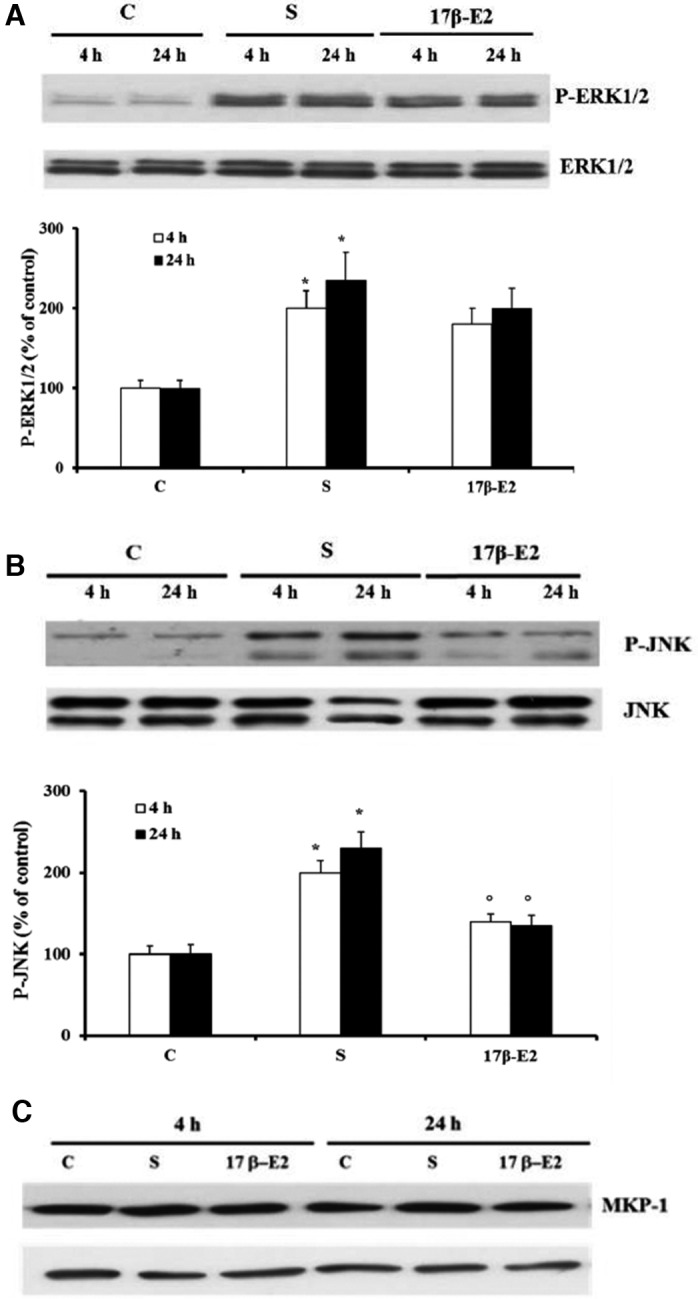
Effect of 17β‐E2 on MAPKs phosphorylation and MKP‐1 expression in MLO‐4Y cells. Phosphorylation of ERK1/2 (A) and JNK (B) and MKP‐1 expression (C) were detected by western blot analysis in MLO‐Y4 cells cultured for 4 or 24 h in complete medium (C, control) or in serum‐free medium (S, starved cells). S were treated or not with 10 nm 17β‐E2 as reported in Materials and methods. Blots are representative of four experiments and the values are normalized with ERK1/2 or JNK bands obtained by densitometric analysis and reported as mean percentage ± SEM relative to the respective C values in the bottom. *P ≤ 0.05 compared to the respective C values; ^○^ *P* ≤ 0.05 as compared to the respective S values.

Figure 3 shows that both ERK1/2 and JNK phosphorylation increased after 4 h and 24 h from starvation, and 17 β‐E2 inhibited JNK activation at both times by about 60–70%, as compared with unstimulated starved cells (Fig. 3B); no significant effect of 17 β‐E2 on ERK1/2 activation was observed (Fig. 3A). Previously, we related starvation‐induced apoptosis to JNK activation 21, therefore, in order to clarify the involvement of JNK inhibition on 17 β‐E2 antiapoptotic effect, the expression of MKP‐1, a specific phosphatase for JNK 32, 33, was investigated. Figure 3C shows no change in MKP‐1 expression at both times in 17 β‐E2 cells, stimulated or not.

### Effect of 17 β‐E2 and NAC on JNK association with monomeric GSTP1‐1 form and GSTP1‐1 expression in starved MLO‐Y4 cells

**Figure 4:**
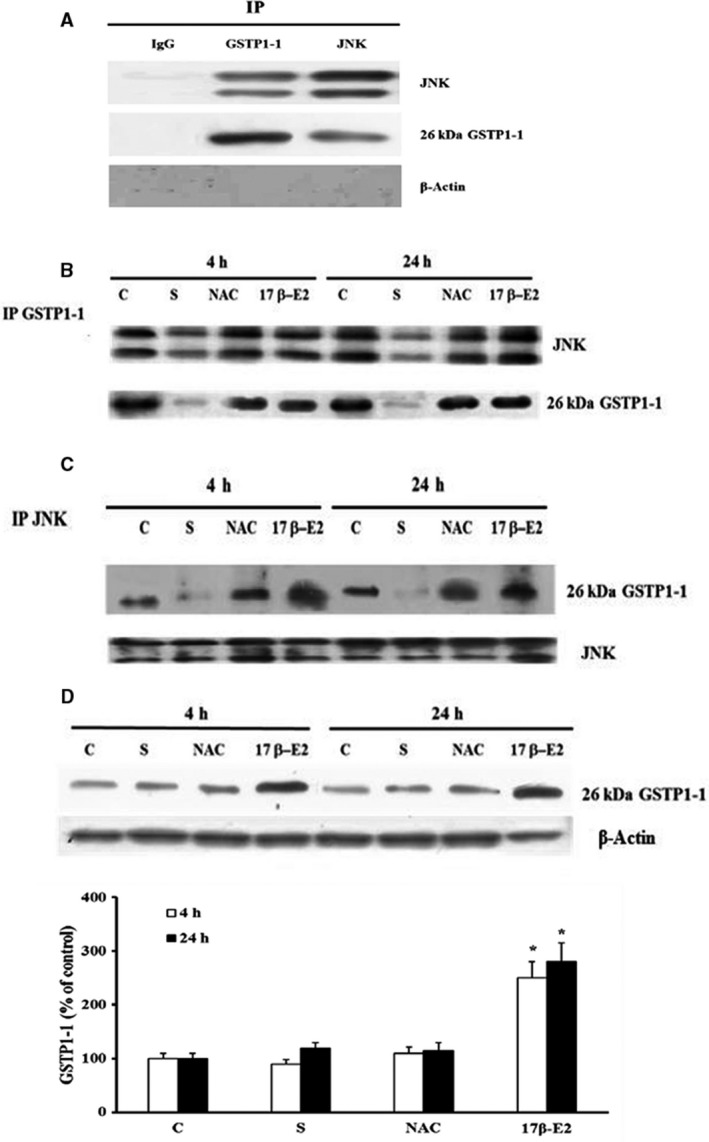
Effect of 17β‐E2 and NAC on JNK association with monomeric GSTP1‐1 form and GSTP1‐1 expression in MLO‐4Y cells. JNK association with GSTP1‐1 and GSTP1‐1 expression were detected in MLO‐Y4 cells cultured for 4 or 24 h in complete medium (C, control) or in serum‐free medium (S, starved cells). S were treated or not with 10 nm 17β‐E2 or 5 mm NAC as reported in Materials and methods. The negative control was performed in MLO‐Y4 cells cultured for 24 h in complete medium by immunoprecipitation with negative control mouse IgG (A). For detection of JNK bound to‐GSTP1‐1 or IgG (A and B), or GSTP1‐1 bound to JNK or IgG (A and C), or βActin bound to IgG (A) all immunoprecipitates of equal proteins (400 μg) were performed using anti‐GSTP1‐1 antibody or anti‐JNK antibody or IgG respectively, as reported in Materials and Methods. Western blot analyses were performed under non‐reducing conditions (without β‐mercaptoethanol) and membranes were probed with anti‐JNK (A and B) or anti GSTP1‐1 (C) antibodies and subsequently, after stripping, with anti‐GSTP1‐1 (A and B) or anti‐JNK (C) or anti‐βActin (A) antibodies, respectively. GSTP1‐1 expression (D) was detected by Western blot analysis performed under reducing condition. The normalized values with β‐Actin bands obtained by densitometric analysis are reported as mean percentage ± SEM relative to the respective C values in the bottom. The blots are representative of four experiments. *P ≤ 0.05 compared to the respective C values.

Previous data demonstrated that GSTP1‐1 monomeric form binds JNK, inhibiting its activation, and the ability of GSTP1‐1 to bound JNK is related to oxidative state and/or to GSTP1‐1 expression level 34, 35, 46, 47. In Fig. 4A no band of JNK or GSTP1‐1 or β‐Actin was detected in Western blot analysis of immunoprecipitates performed with IgG (negative control) in control cells. Differently, JNK and GSTP1‐1 bands and no β‐Actin were detected after immunoprecipitation with anti‐GSTP1‐1 or anti‐JNK antibody (Fig. 4A). These data demonstrate the absence of non‐specific bands under these experimental conditions. Figure 4B shows JNK bands obtained by western blot analysis under nonreducing SDS/PAGE of immunoprecipitates performed with anti‐GSTP1‐1 antibody; a decrease in JNK associated with monomeric GSTP1‐1 form (26 kD) was registered only in starved cells as compared with control. On the contrary, both 17 β‐E2 and NAC treatments prevented this dissociation after 4 and 24 h, and the band density of JNK was similar to those of controls (Fig. 4B). The stripping and reprobing of the same blots by anti‐GSTP1‐1 antibody show that the band density of GSTP1‐1 monomeric form changed similarly to that of JNK bands (Fig. 4B). The same results with regard to the association of monomeric form of GSTP1‐1 with JNK were obtained by JNK immunoprecipitation experiments performed under nonreducing SDS/PAGE (Fig. 4C).

To evaluate the expression of total GSTP1‐1 in 17 β‐E2‐ and NAC‐treated cells, western blot analysis of cellular lysates in reducing conditions was performed (Fig. 4D). A significant increase in total GSTP1‐1 expression in estrogen‐treated cells, as compared to control, was measured but no variation was noted in starved and NAC‐treated cells.

### Role of GSTP1 in 17 β‐E2 and NAC effect on apoptosis and JNK activation in starved MLO‐Y4 cells

**Figure 5:**
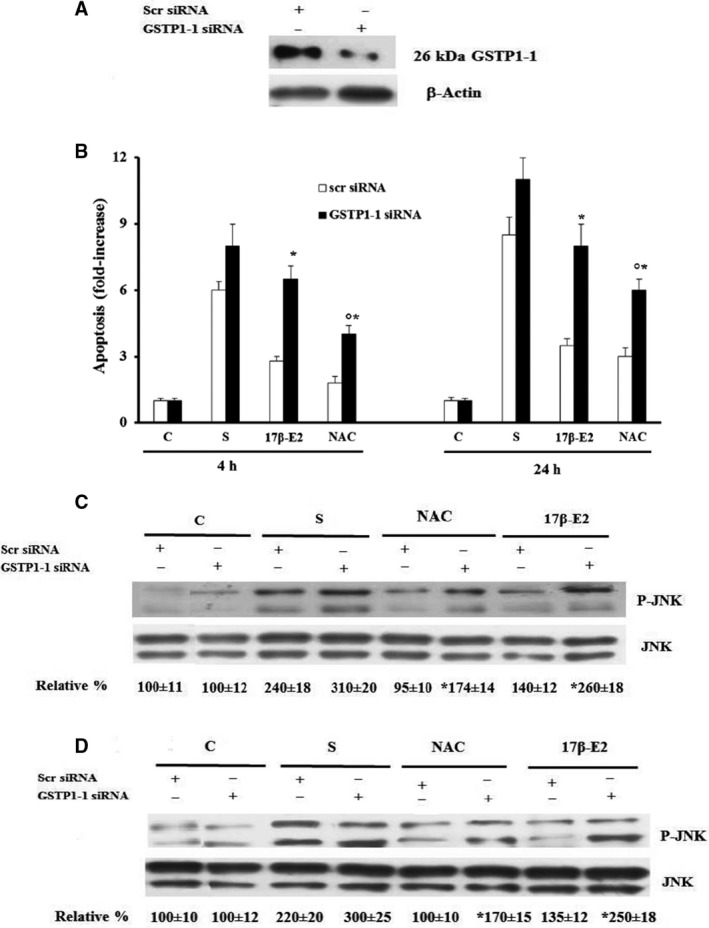
Effect of GSTP1‐1 siRNA on apoptosis and JNK phosphorylation in 17β‐E2 or NAC treated MLO‐4Y cells. MLO‐Y4 cells, transfected with GSTP1‐1 siRNA or scr siRNA (negative control) for 24 h, were cultured for 4 or 24 h in complete medium (C, control) or in serum‐free medium (S, starved cells). Transfected S were treated or not with 10 nm 17 β ‐E2 or 5 mm NAC as reported in Materials and methods. GSTP1‐1 expression (A) and JNK phosphorylation after 4 h (C) or 24 h (D) were detected by western blot analysis. The blots are representative of three experiments and the normalized values with JNK bands obtained by densitometric analysis are reported as mean percentage ± SEM relative to the respective C values. Apoptosis data (B), relative to mono‐ and oligonucleosomes released into the cytoplasmic fraction from 10^4^ cells, are expressed as fold‐increase over C values, and are the mean ± SEM of three independent experiments. *P ≤ 0.05 compared to the respective scr siRNA‐treated cell values. ^○^ *P* ≤ 0.05 compared to scr siRNA S values.

Figure 5A shows that GSTP1‐1 expression decreased in control cells transfected with specific siRNA after 24 h of transfection. Figure 5B shows that the down‐regulation of GSTP1‐1 induced a significant increase in apoptosis in starved cells treated with NAC or 17 β‐E2 at both studied times, as compared to the respective scr siRNA‐starved cell values (Fig. 5B). In particular, down‐regulation of GSTP1‐1 in 17 β‐E2‐treated cells reverted the apoptosis and JNK activation levels to the values measured in untreated scr siRNA starved cells (Fig. 5B–D). In NAC‐treated cells, down‐regulation of GSTP1‐1 increased also apoptosis and JNK activation levels, but these did not reach the levels of untreated scr siRNA starved cell, even if the changes in JNK activation were not significant as compared with untreated scr siRNA‐starved cells (Fig. 5B–D).

**Figure 6:**
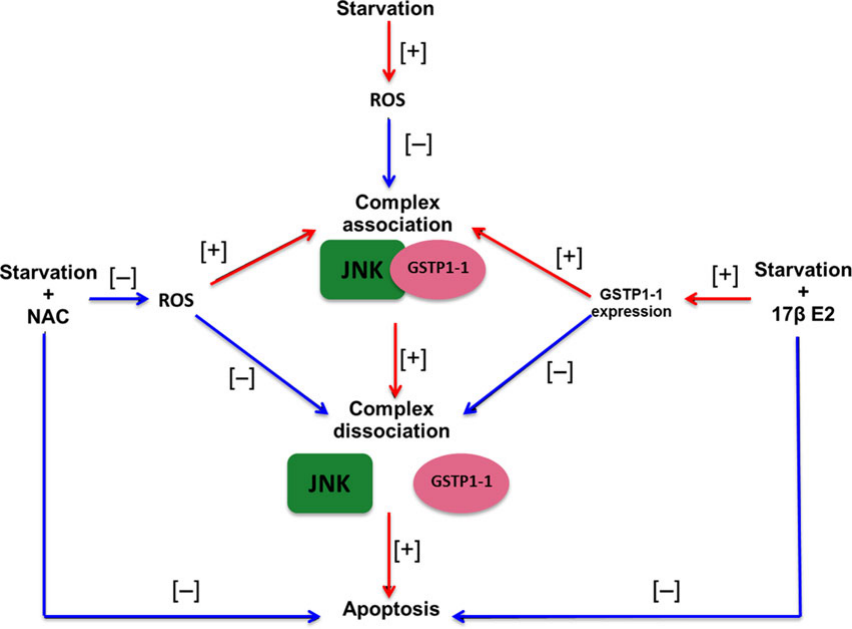
Summary of 17β‐E2 and NAC different mechanisms on JNK‐GSTP1‐1 complex formation in starved MLO‐Y4 cells. NAC and 17β‐E2 reduce JNK‐GSTP1‐1 complex dissociation‐ and starvation‐induced apoptosis favoring JNK/GSTP1‐1 complex association by ROS decrease and GSTP1‐1 expression increase, respectively. [+] = increase; [−] = decrease

Figure 6 summarizes the possible effects of 17 β‐E2 and NAC on the association of JNK to GSTP1‐1 in relation to JNK and apoptosis activation in starved MLO‐4Y cells.

### Effect of 17 β‐E2 on expression and release of RANKL and OPG, and on Sclerostin expression in starved MLO‐Y4 cells

**Figure 7:**
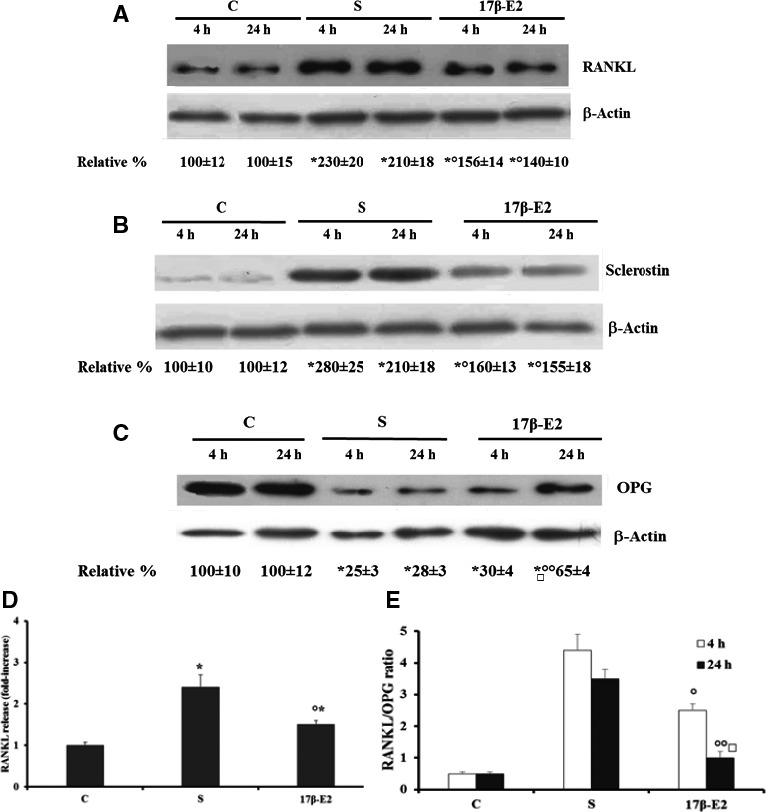
Effect of 17β‐E2 on RANKL, sclerostin, and OPG expression and RANKL release in MLO‐Y4 cells. RANKL, sclerostin, and OPG expression and RANKL release were measured in MLO‐Y4 cells cultured for 4 or 24 h in complete medium (C, control) or in serum‐free medium (S, starved cells). S were treated or not with 10 nm 17β‐E2 as reported in Materials and methods. RANKL (A), sclerostin (B), and OPG (C) were measured by western blot analyses of cell lysates. Blots are representative of four experiments and the normalized values with β‐Actin bands obtained by densitometric analysis are reported as mean percentage ± SEM relative to the respective C values. RANKL release (D) was detected by immunoenzymatic method in 24 h culture medium, and the values are expressed as fold‐increase over the control values. (E) RANKL/OPG ratio was obtained by the expression values measured with western blots. The data are the mean ± SEM of four independent experiments. *P ≤ 0.05 compared to the respective C values; ^○^ *P* ≤ 0.05 and ^○○^ *P* ≤ 0.005 compared to the respective S values; ^□^ *P* ≤ 0.005 compared to S values for 4 h and stimulated with 17β‐E2.

The expression of RANKL, OPG, and sclerostin was determined in starved MLO‐4Y cells treated with 17 β‐E2, considering that in these cells a relationship among apoptosis, increased oxidative state, and expression of these factors has been previously found 21. In particular, in the cited study, in starved MLO‐Y4 cells oxidative stress‐induced activation of JNK and ERK1/2 and this was related to RANKL and sclerostin up‐regulation, whereas only JNK activation was involved in decreased OPG levels 21. Figure 7A,B shows, after 4 or 24 h of starvation, a significant increase in RANKL and sclerostin levels, as compared with control, and 17 β‐E2 treatment decreased them by about 50%‐60%, but levels did not return to those of controls. On the contrary, OPG remarkably decreased at both starvation times, as compared with control cells, and 17 β‐E2 inhibited by about 50% the reduction of OPG expression only after 24 h of starvation (Fig. 7C). Also, in this case estrogen was not able to revert OPG levels to the control value (Fig. 7C). Figure 7D shows that 17 β‐E2 treatment significantly lowered RANKL release after 24 h of starvation as compared with untreated and starved cells. However, 17 β‐E2 did not restore RANKL release to control values in accordance to that observed on RANKL expression. Similar results were obtained after 4 h of starvation (data not shown). Regarding OPG protein release, we were not able to measure the levels of this protein by ELISA kit in MLO‐Y4 in agreement with results previously reported by us and others 21, 31. For this reason, the RANKL/OPG ratio was measured by the expression values obtained in western blot analysis (Fig. 7A,C). The ratio increased in a similar manner after 4 h and 24 h of starvation as compared with control cells (Fig. 7E), and 17 β‐E2 significantly lowered the ratio values at both times by about 40–70%, respectively (Fig. 7E). The decrease in ratio value after 24 h of starvation was significantly higher than that obtained after 4 h.

## Discussion

This study provides new evidence that 17β‐E2 in starved MLO‐Y4 quickly reduces apoptosis and the related caspase‐3 activation due to increased oxidative state, without significant inhibition of ROS production. Under the same experimental conditions, 17β‐E2 prevents the increase in RANKL and sclerostin expression, RANKL release, and RANKL/OPG ratio. For the first time, these effects of 17β‐E2 are shown to occur by a redox‐independent process of JNK activation involving GSTP1‐1 overexpression. The ability of 17β‐E2 to prevent starvation‐induced apoptosis is similar to that obtained in MLO‐Y4 after treatment with the antioxidants 21, indeed, we previously demonstrated a redox‐regulated osteocyte apoptosis due to starvation. However, our data in MLO‐Y4 demonstrate that 17β‐E2 significantly decreases starvation‐induced apoptosis and caspase‐3 activation at both the studied times, but only after 24 h from the starvation does 17β‐E2 show a weak antioxidant action. On the contrary, NAC, a direct ROS scavenger, remarkably reduces ROS levels at both times, and this has previously been related to its ability to prevent starvation‐induced osteocyte apoptosis 21. Some authors demonstrate that the antiapoptotic effect of estrogen is due to its action as direct ROS scavenger 14 and others indicate 17β‐E2 as inhibitor of NADPH oxidase 43. However, our findings do not show a relationship between the antiapoptotic effect of 17β‐E2 and its antioxidant action, which seems to be weak, late, and caused by the inhibition of NADPH oxidase activity, a membrane enzyme which increases cytoplasmic oxidative state 44. Diversely, in starved osteocytes the increased ROS levels and consequent apoptosis at both times seem to be mainly of mitochondrial origin, and this agrees with previous data which show in MLO‐Y4 the involvement of JNK activation 21. Indeed, JNK activity prevalently mediates stress‐induced apoptosis by mitochondrial pathways 48, and this may occur in osteocytes through increased permeability of the outer mitochondrial membrane with the release of cytochrome C‐activating caspases (caspase‐3,‐6 and ‐7) responsible for cell death 19.

Effectively, it seems difficult for estrogen to have a direct antioxidant effect as shown by others who induced apoptosis by extracellular H_2_O_2_ treatment in MLO‐Y4 cells 14. In fact, in this study, the estrogen was used at a much lower concentration (10 nm) than that of H_2_O_2_ (0.3 mm). Also “*in vivo*” it is unlikely that estrogens may have a direct antioxidant effect given that their circulating levels are lower than the concentrations of chemical antioxidants necessary to reduce the oxidative stress 9, 49. Indeed, “*in vitro*” and “*in vivo*” studies demonstrate that 17β‐E2 antioxidant action is related to an up‐regulation of antioxidant enzymes 9, 11, 12, 13, 49. However, this mechanism may require longer than a direct antioxidant action, and our data suggest that the early antiapoptotic effect of 17β‐E2 occurs in the presence of an oxidative state. As previously reported, JNK and ERK1/2 are activated by an increased oxidative status in starvation‐induced osteocyte apoptosis 21. However, it has been demonstrated that only JNK activity is involved in oxidative stress‐induced apoptosis in MLO‐Y4, and that the antioxidants including NAC are able to inhibit both JNK and ERK1/2 activity by decreasing ROS levels 21. Similarly to antioxidants, 17β‐E2 inhibits JNK activation in starved MLO‐Y4 cells at both studied times, and this can be related to the antiapoptotic effect of 17β‐E2, but not to decreased ROS levels. This indicates that 17β‐E2 antiapoptotic effect occurs through a process which is unrelated to oxidative status. Moreover, 17β‐E2 does not affect ERK1/2 activation due to the presence of the oxidative status, indicating that ERK1/2 activation is not involved in the 17β‐E2 protective effect against osteocyte apoptosis, similarly to that observed by others in osteocyte apoptosis induced by H_2_O_2_ treatment 14.

No data are reported in literature on a protective effect of 17β‐E2 on oxidative stress‐induced apoptosis in osteocytes by down‐regulation of JNK activity. For this purpose, we studied the role of MKP‐1, a member of the MKP family that predominately dephosphorylates JNK 32, 33, in estrogen‐induced JNK inactivation. Indeed, through its receptors 17β‐E2 can induce genomic expression of MKP‐1 in various cells including bone cells 32, and it has been shown that MKP‐1‐modulated JNK activity is critical for apoptosis induced by the inhibition of epidermal growth factor receptor tyrosine kinase in lung cancer cells 32. We demonstrated that MKP‐1 is expressed in osteocytes but, under the studied experimental conditions, 17β‐E2 and the increased oxidative state do not induce an overexpression of this phosphatase, excluding its possible role in JNK inactivation by estrogen.

Our findings demonstrate, for the first time, that GSTP1‐1, a detoxifying enzyme also involved in cell death and/or survival mechanisms, is expressed in osteocytes and is related to the antiapoptotic effect of 17β‐E2 mediated by JNK inactivation in the presence of oxidative state. In fact, it is well demonstrated that GSTP1‐1 is a regulator of JNK signaling pathways through the formation of a complex with c‐Jun‐JNK. This association occurs with GSTP1‐1 monomeric form and selectively inhibits the phosphorylation and activation of JNK 34, 35, 46, 47, 50. The present work demonstrates that, under normal physiological conditions, GSTP1‐1 monomeric form is in reversible equilibrium with dimeric and polymeric forms and that this balance shifts, in the presence of oxidative state, versus polymeric forms due to the formation of disulfide bonds 47, 48, 50, inducing GSTP1‐1/JNK dissociation and apoptosis activation 34, 35, 46, 47. In agreement with literature data, immunoprecipitation and immunoblot experiments demonstrate that in MLO‐4Y cells JNK is bound to GSTP1‐1 and under conditions of oxidative stress both the levels of bound JNK and GSTP1‐1 monomeric form decrease in starved cells. This is inversely related to JNK activation which increases only when there are high levels of ROS, suggesting GSTP1‐1/JNK dissociation. This redox regulation of GSTP1‐1/JNK complex formation is confirmed by findings obtained with NAC treatment. In fact, NAC preserves JNK bound levels, preventing complex dissociation and JNK activation. This could be due to the ability of NAC to preserve high levels of GSTP1‐1 monomeric form through the lowering of ROS levels and the breaking of disulfide bonds present in GSTP1‐1 oligomeric forms.

Our data show that 17β‐E2 is also able to maintain high levels of both GSTP1‐1 monomeric form and JNK linked to GSTP1‐1. This indicates that estrogen may preserve GSTP1‐1/JNK complex dissociation with the consequent JNK inactivation even if an oxidative state is present. The effect of 17β‐E2 can be related to the overexpression of GSTP1‐1, which is consistent with data showing that the overexpression of GSTP1‐1 can affect the balance between the various forms of GSTP1‐1 and the possible bond with JNK 34, 35, 46, 47, 51. Indeed, the increased GSTP1‐1 expression can promote the formation of the GSTP1‐1/JNK complex by counteracting the action of ROS which, differently, induce complex dissociation and JNK activation. This has been demonstrated by other researchers who have correlated it to apoptosis resistance of tumor cells 34, 46, 47. In agreement with our data, Bartolini *et al*. 51 report that GSTP1‐1 overexpression inhibits stress‐induced JNK activation, whereas it does not affect ERK1/2 activation.

Direct involvement of GSTP1‐1 in the protective effect of 17β‐E2 and NAC on oxidative stress‐induced apoptosis and on their ability to modulate JNK activation has been shown by GSTP1‐1 down‐regulation. In particular, GSTP1‐1 seems important in mediating the early antiapoptotic effect of estrogen through inhibition of JNK in the presence of an oxidative state. In fact, in 17β‐E2‐treated cells, down‐regulation of GSTP1‐1 similarly reverts both apoptosis and JNK activation to starved cell values. Also in NAC‐treated cells, down‐regulation of GSTP1‐1 increased both apoptosis and JNK activation levels, but these do not return to starved cell values, indicating that stress‐induced JNK activation, followed by apoptosis, may also in part depend on other GSTP1‐1‐independent redox‐regulated factors.

Previously, we demonstrated that when there is an oxidative state in MLO‐Y4 the expression of factors, such as RANKL, OPG, and sclerostin involved in bone remodeling, are related to apoptosis as well as to JNK and ERK1/2 activity 21. Indeed, these factors are expressed by osteocytes under various conditions, including bone pathological alterations 1, 2, 3, 4, 7, 11, 52, 53. RANKL increases osteoclast differentiation and bone resorption, whereas OPG competes with RANKL for its receptor inhibiting osteoclastogenesis 2, 3, 4, 52, 53. Moreover, it has been shown that regulation of the RANKL/OPG ratio is one of the means by which bone resorption and formation can be maintained in equilibrium, and the RANKL/OPG ratio is indicative of osteoclastogenic activity in various bone remodeling diseases 1, 3, 52, 53. Sclerostin (mainly produced by mature osteocytes) is a negative regulator of osteoblast activity and OPG production 1, 2, 3, 4, 5, 53 and it inhibits the Wnt/beta‐catenin signaling pathway preventing OPG production 1, 2, 3.

17β‐E2 is able to prevent the increased expression and release of RANKL and the increased expression of sclerostin. However, 17β‐E2 is not able to completely revert RANKL and sclerostin levels to those of control, unlike antioxidants which in these cells inhibit both JNK and ERK1/2 activity 21. 17β‐E2 is also able to prevent, in part, the remarkable decrease in OPG levels observed in starved osteocytes, similarly to antioxidants, as only JNK activity is involved in the down‐regulation of OPG expression 21. Given that both estrogen and antioxidants almost totally inhibit JNK activity, their inability to restore normal levels of OPG expression can be correlated with the involvement of other JNK‐ and/or oxidative stress‐independent factors and likely related to starvation. All these data show that the 17β‐E2 effect on expression of the studied cytokines is mainly mediated by JNK inhibition. 17β‐E2 remarkably decreases RANKL/OPG ratio after 24 h from starvation because only at this time 17β‐E2 significantly increases OPG levels. However, 17β‐E2, different from that which occurs with antioxidants, does not revert the ratio values to those of the control 21 probably because of the inability of estrogen to quickly eliminate ROS and to inhibit ERK1/2 activation which affects RANKL expression. All these data show that the fast effect of 17β‐E2 on the expression of the studied cytokines in the presence of oxidative state depends mainly on GSTP1‐1 expression that affects JNK activity. Thereafter, the regulation of GSTP1‐1/JNK association may be related to estrogen signaling and RANKL/OPG levels.

These data in osteocytes support the efficacy of estrogen treatment in postmenopausal women by decreasing sclerostin and RANKL levels, which result enhanced in serum during estrogen deficiency 1, 2, 54. Indeed, both high levels of RANKL and sclerostin are implicated in the increased bone resorption associated with estrogen deficiency and microdamage 12, 19, 22, 23, 52. The effect of 17β‐E2 on the up‐regulation of OPG may be related to the down‐regulation of sclerostin expression, and this event may be also related to the modulation of JNK activity by up‐regulation of GSTP1‐1. This agrees with a potential crosstalk between the Wnt/beta‐catenin signaling pathway and estrogen signaling 3, 4, 37.

## Conclusions

The present work shows, for the first time in MLO‐Y4 osteocyte‐like cells, that 17β‐E2 prevents early stress‐induced osteocyte apoptosis which can occur “*in vivo*” in estrogen deficiency by inhibition of JNK activation due to an increased oxidative state. A novel and important direct involvement of GSTP1‐1 in the 17β‐E2 effect on JNK activation and apoptosis in osteocytes has been demonstrated. 17β‐E2 up‐regulates GSTP1‐1 expression, contributing to maintain JNK bound to GSTP1‐1 and then to inhibit JNK activity. Our study also shows that estrogen achieves an effect similar to NAC that, through oxidative state elimination, is able to inhibit apoptosis and JNK activity while maintaining JNK bound to GSTP1‐1. In fact, others have demonstrated that the formation of GSTP1‐1/JNK complex may be regulated by oxidative state changes and/or GSTP1‐1 overexpression. It is interesting to note that the antiapoptotic effect of 17β‐E2 is not due to a direct and quick antioxidant action. Indeed, antioxidant activity of estrogen can occur later through the increased expression of antioxidant enzymes as other researchers have demonstrated. Moreover, this study indicates that 17β‐E2 through JNK inhibition by GSTP1‐1 association can decrease the RANKL/OPG ratio and sclerostin level, factors related to osteogenesis and bone remodeling.

Overall, the data presented here clarify 17β‐E2 action at molecular level in osteocyte activity on bone remodeling, and identify a possible role of GSTP1‐1 and JNK activity in bone repair mechanisms in pathologies related to oxidative stress. Finally, they confirm the validity of antioxidants as therapeutic support.

## Author contribution

MTV and MLB performed the design of the study, interpreted the results, and participated in the writing and drafting manuscript; FF and VD performed experiments and collected data; GM and TI participated in the design of the study and performed statistical analysis; MTV, MLB, FF, VD, GM, and TI revised manuscript content and provided final approval of the manuscript version to be published.
